# Can a lexical decision task predict efficiency in the judgment of ambiguous sentences?

**DOI:** 10.1186/s41155-018-0093-0

**Published:** 2018-06-15

**Authors:** Paulo Guirro Laurence, Tatiana Matheus Pinto, Alexandre Tadeu Faé Rosa, Elizeu Coutinho Macedo

**Affiliations:** 1 Social and Cognitive Neuroscience Laboratory, Center for Health and Biological Sciences, Mackenzie Presbyterian University, Rua Piaui, no. 181, 10th floor, São Paulo, 01241-001 Brazil; 2 Developmental Disorders Program, Center for Health and Biological Sciences, Mackenzie Presbyterian University, São Paulo, Brazil

**Keywords:** Reading, Comprehension, Semantic decision, Eye-tracking, Ambiguity, Regressive saccades, Lexicon

## Abstract

The lexicon plays a fundamental role in reading, but little is known about how it influences reading efficiency. Thus, this study seeks to identify which lexical factors in a lexical decision task are relevant in a semantic decision test. A total of 33 university students were recruited to perform a lexical decision task and a semantic decision task. The results revealed differences between the three types of words in the lexical decision task for all measures, but only in the regressive saccades for the semantic decision task. Ambiguous sentences triggered fewer regressions than sentences related to objects. The only lexical measure found to predict efficiency was average time on regular words, which predicted 24% of the efficiency. We discuss the implications of the use of a lexical decision task and the use of the inverse efficiency score as a semantic measure, and we discuss how the lexicon can predict semantic comprehension.

## Background

The ability to read is essential for knowledge acquisition because of the increasing importance of formal education. Reading also plays a key role in human communication and has profound implications for human cognitive development (Cunningham & Stanovich, 1998).

The process of reading has several components, including the lexical component and the semantic component (Gazzaniga, Ivry, & Mangun, 2002). The lexical component is responsible for the systematic organization of vocabulary and the storage of word-related information for word recognition and comprehension (Fernald, Perfors, & Marchman, 2006; Lupker, 2011). This information includes the phonology, morphology, and semantics of words (Field, 2004) and is also linked to reading comprehension (Perfetti & Stafura, 2014).

Lexical decision tasks are used to evaluate lexical access and lexical formation. They enable the analysis of lexical items (Gijsel, Bon, & Bosman, 2004), which can be either real words or pseudo-words (Balota & Chumbley, 1984). Lexical decision tasks allow the mapping of orthographic processing at two different levels. First, they can be used to compare the sensitivity of visual stimuli with letters and stimuli with graphic images unrelated to written language. Second, they enable a contrast between familiar and non-familiar spelling items. These items can reveal subjects’ familiarity with orthographic representations as well as the level of development of their visual lexicon (Hasko, Groth, Bruder, Bartling, & Schulte-Körne, 2013). These types of tasks have been used in a range of studies, from those examining memory (Hicks, Franks, & Spitler, 2017) to event-related potentials (Araújo, Faísca, Bramão, Reis, & Petersson, 2015; Haro, Demestre, Boada, & Ferré, 2017). While widely used, however, these tasks involve a high degree of noise (Diependaele, Brysbaert, & Neri, 2012) and may not be the best tools for measuring lexical access (Balota & Chumbley, 1984). Rayner and Liversedge (2013) also note that lexical decision tasks may reflect an oversimplification of the reading process.

Meanwhile, the semantic component is linked to the comprehension of words and sentences, whose meanings are connected and form a complex network that gives meaning to the text (Kintsch & Rawson, 2011).

Comprehension studies use a garden path model, a paradigm that accounts for the reading and comprehension of ambiguous sentences. According to this model, ambiguous sentences can be viewed as structurally analogous to “garden paths” with nodes joining multiple branching paths, and we tend to interpret these sentences through the path with fewest nodes (Frazier, 1987; Frazier & Rayner, 1982; Gazzaniga et al. 2002). Thus, experiments on comprehension typically focus on the number of correct items and the reaction time for each participant. Little evidence has emerged on the differences found by these measures for processing ambiguous or unambiguous sentences, although processing ambiguous items involves an extra cost (Clifton, Staub, & Rayner, 2007).

Researchers have used different tasks to evaluate reading comprehension. Although all tasks measure the same elements in principle, the values of these tests often do not correlate, which suggests they may not measure the same skills (Keenan, Betjemann, & Olson, 2008). What most tests have in common is that they measure accuracy percentage (or the number of errors) and time. However, non-intrusive measures, such as ocular movement analyses, may improve the understanding of text comprehension by measuring eye movements such as fixations, average fixation duration, first fixation duration, gaze duration, skipping rates, and regression rates (Juhasz & Pollatsek, 2013). These factors can reveal critical real-time information about reading comprehension. Studies have shown, for example, that when readers find sentence comprehension difficult, they perform inter-word regressions (Frazier & Rayner, 1982; Vitu, 2013). Thus, eye-tracking is important for reading comprehension studies.

Some studies have attempted to use the lexicon to predict semantic performance. For instance, Swart et al. (2017) measured several variables related to the lexicons of fourth-grade students and attempted to predict the students’ outcomes from a general mean consisting of several semantic tasks. The authors were able to predict 65% of the score, but only 30% of this variance was related to the lexical component. Hence, only a limited amount of semantic comprehension can be predicted from lexical factors. Ouellette (2006) found similar results: This author was able to predict 28.5% of the variance in reading comprehension among a group of fourth-grade students with vocabulary measures. Cutting and Scarborough (2006) were able to predict 6.1 to 11.9% of comprehension measures in first- through tenth-grade students through their word recognition/decoding skills. Additionally, lexical factors have been successfully used to predict reading development (Verhoeven, van Leeuwe, & Vermeer, 2011) as well as some, but not all, reading skills (Ricketts, Nation, & Bishop, 2007).

To clarify the relationship between the lexical and semantic components and laboratory tests for these skills, this study aims to identify the lexical factors in lexical decision tasks relevant for semantic decision tests. To this end, we designed two tasks (including eye-tracking analyses for the semantic task) to predict the efficiency of the semantic task.

## Methods

### Participants

A total of 33 university students (22 women; age M = 22.2, S.D. = 3.29) participated in the study. These participants were all right-handed and had normal or lens-corrected vision, no diagnosis of psychiatric or neurological disorders, and no school attendance issues. The number of participants was calculated considering an alpha of 5%, beta of 90%, and effect size of 0.26 (large). The calculation was made on G*Power® 3.1.9.2 (Buchner, Erdfelder, Faul, & Lang, 2017). The participation of all subjects was voluntary and approved by the university’s Research Ethics Committee. Subjects gave written informed consent and received course credit in return at the end of the procedure.

### Adult Dyslexia Checklist

The Adult Dyslexia Checklist (ADC; Vinegrad, 1994) is a questionnaire of 20 items, all of which are related to symptoms of different areas of dyslexia. The items comprise questions in a “yes” or “no” answer format (e.g., “Is map reading or finding your way to a strange place confusing?”). For each item marked in the affirmative, a point is added to the test result.

Although the instrument may indicate the possibility of dyslexia, it is not a diagnostic tool. In other words, the data collected in this test are not sufficient to definitively identify dyslexia. However, the test results have a high indicative value for dyslexia. It would be useful to suggest that subjects with high scores undergo an evaluation with a complete multidisciplinary team (Vinegrad, 1994).

### Lexical decision task

The lexical decision task was adapted from Oliveira (2014). We incorporated the feasibility criteria for the application and recording of behavioral responses and ocular movements. Three categories of linguistic items were defined, yielding a total of 216 items: 72 regular words, 36 pseudo-words, and 108 quasi-words. The syllabic structure of the stimuli was counterbalanced among CVCVCV (e.g., *Pirata* [Pirate]), VCVCV (e.g., *Urina* [Urine]), CCVCVCV (e.g., *Granada* [Granada]), and VCCVCV (e.g., *Osmose* [Osmosis]) structures. The number of letters in the stimuli ranged between 5 and 7 letters, so length had no influence on the processing of the items.

All words used have a medium or high frequency of use in Portuguese, according to the NILC Corpus of the University of São Carlos (http://www.linguateca.pt/ACDC/). We selected words with regular structures and rules. Quasi-words comprised three subtypes of pseudo-words (e.g., Seabra, Dias, Mecca, & Macedo, 2017): quasi-words with visual exchanges, quasi-words with phonological exchanges, and quasi-words with pseudo-homophones. The criteria for the classification of these quasi-word subtypes have been supported in the literature on cognitive models of reading, since errors in the reading of irregular words indicate difficulties in, or the absence of lexical processing (Ellis & Young, 1988). Our categorization is based on that used by Proverbio and Adorni (2008).

Pseudo-words were constructed of sequences of decodable letters and syllables but not derived from real words. For this reason, the frequency values of the bigrams of the task stimuli with 5 and 6 letters were measured according to Justi and Justi (2009).

**Fig. 1:**
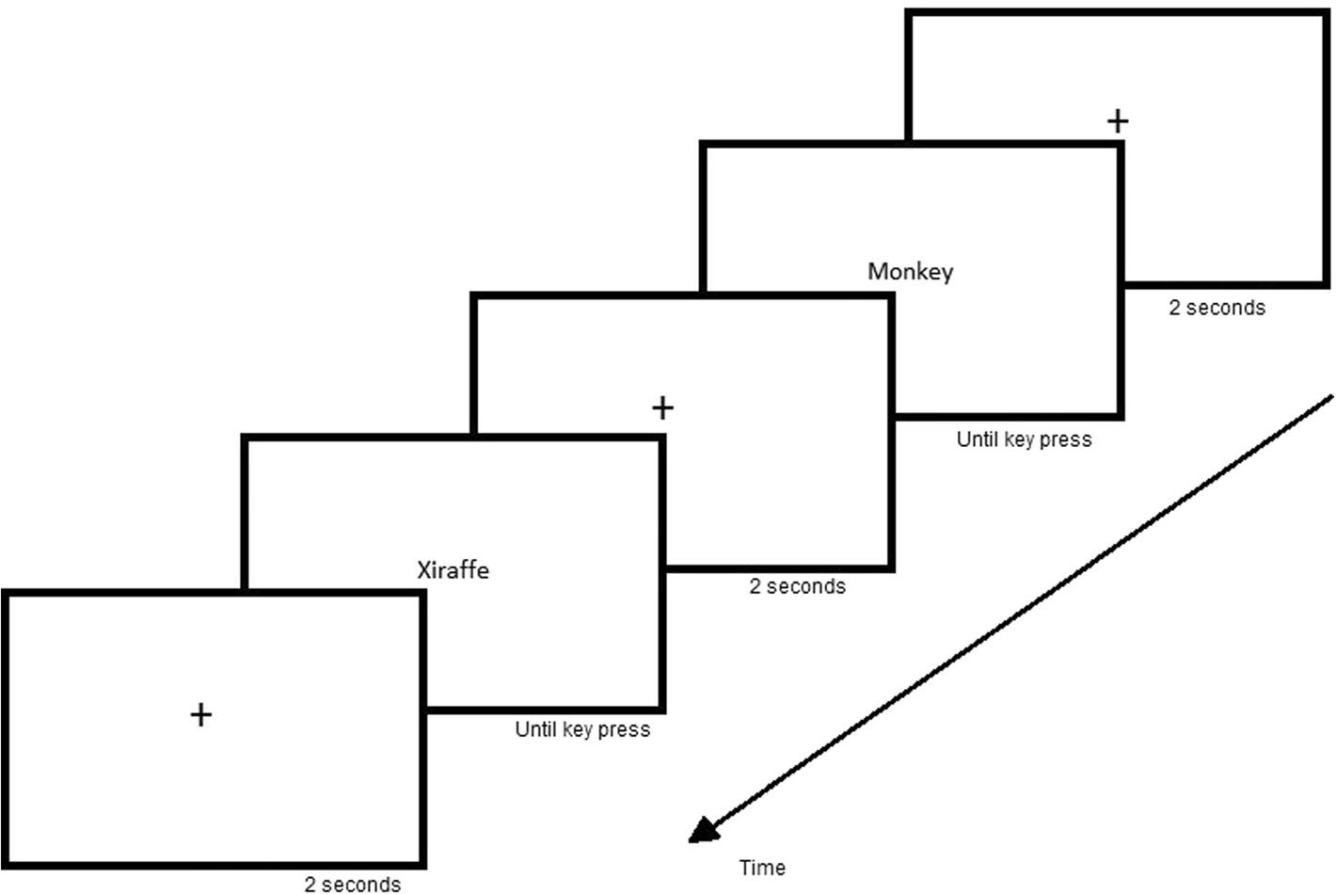
Experimental design for lexical decision task. The stimuli were presented in Portuguese

The task stimuli were created as Joint Photographics Experts Group (JPEG) files with a resolution of 1280 × 720 pixels. The font used was 22-point Calibri in black on a white background. Between each word presented, a fixation point was shown for 2 s (see Fig. 1). The order of the words was randomized.

The participants were instructed to judge whether the word was real and to press the letter “Q” on the keyboard with the left hand if so or “P” with the right hand if not. In front of these letters were marks indicating what the keys meant. Participants were instructed to respond as quickly as possible. Only the behavioral data were used in this research.

### Semantic decision task

The semantic decision task was structured to evaluate participants’ ability to judge the ambiguity of written sentences. The task comprised 80 sentences, of which 40 were ambiguous phrases (AMB) and 40 were direct phrases (i.e., unambiguous phrases). Of the direct phrases, 20 were unambiguous sentences with actions related to the subject (ARS) and 20 were unambiguous sentences with actions related to the object (ARO). The sentences had two parts: a first sentence, which gave the context (e.g., “The principal accused the student”), and a second sentence containing the ambiguity or the relation to the subject/object (e.g., “He was processed/He was fired/He was suspended”). The sentences were structured to be the same size with the same number of words (e.g., “The spider attacked the snake. It was poisonous/The spider attacked the snake. It had legs”).

**Fig. 2:**
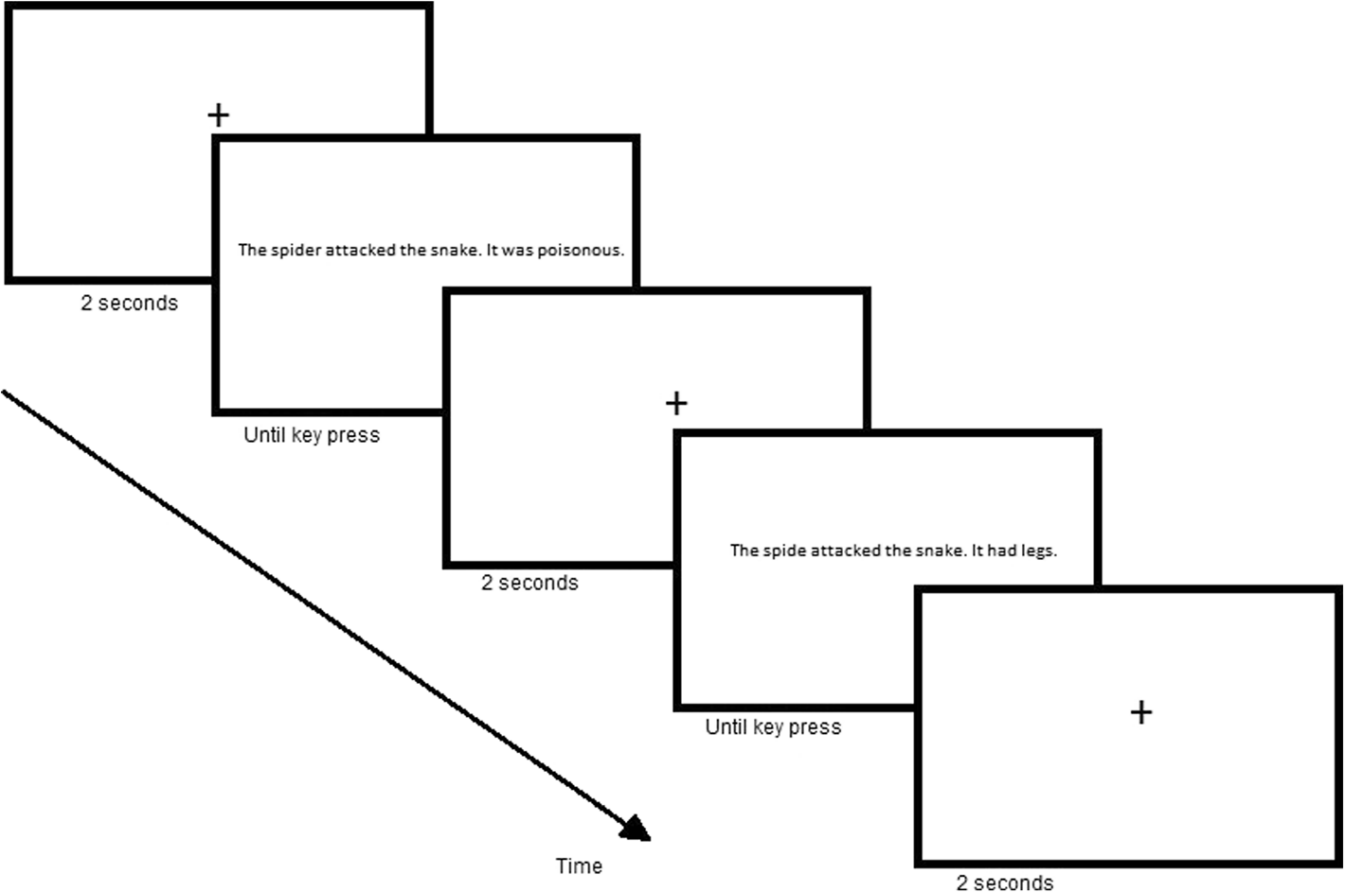
Experimental design for semantic decision task. The stimuli were presented in Portuguese

The task stimuli were created in JPEG files with a resolution of 1280 × 720 pixels. The font used was 22-point Calibri on a white background. The stimuli were presented with intervals of 2 s between the participant’s decision and the display of the next sentence. During this interval, a fixation point was presented at the center of the screen (see Fig. 2). The order of the sentences was randomized.

The instructions given to the participants were similar to those for the lexical decision task. The participants were to judge whether the phrase was ambiguous and to press “Q” on the keyboard with the left hand if so or “P” with his right hand if not. In front of these letters were marks indicating what the keys meant. Participants were instructed to react as quickly as possible.

### Apparatus

The ocular measurement equipment used was the SensoMotoric Instruments (SMI) RED500 (2014). This equipment, which was connected to a 22″ monitor, allowed the measurement of eye movements. Some of the measures that could be obtained with this equipment were the number of fixations, the total fixation time, the number of saccades, the total time in the trial, and qualitative analyses of ocular patterns, among several others.

The device came with experiment development software, SMI Experiment Center ™, and eye movement analysis software, SMI BeGaze™. It was also compatible with third-party software such as E-Prime, which we used to perform the two experiments. Data collection was performed at 500 Hz. The criteria for identifying fixation and saccades were defined as the default in the SMI BeGaze™ version 3.7.104.

### Procedure

The participants came to the laboratory, and the consent terms were explained before they decided whether or not to participate in the research. If they accepted the terms, they completed the ADC and were taken to the room with the eye-tracking equipment. They sat approximately 70 cm from the monitor, which was adjusted to accommodate their physical characteristics. After the participants were positioned, we calibrated the equipment, and the participants then began their first task. The order of the tasks was randomized. For both tasks, participants were given the instructions and started the test when they felt ready. When they had made their judgments about the words or sentences, they pressed the appropriate key on the keyboard in front of them on the monitor table. Between each stimulus presentation, a fixation point was presented at the center of the screen for 2 s. After the test ended, participants received course credit.

### Measures

We assessed several variables in this study. These included the percentage of correct items, the average trial time (in microseconds), and the inverse efficiency score (IES), which is the trial time divided by the correct percentage. This latter variable allows the equalization of the time and correct item percentage. Low scores indicate higher efficiency, and higher scores indicate lower efficiency (Bruyer & Brysbaert, 2011). Other variables were the average number of fixations on trial, the average time per fixation (in microseconds), and the percentage of regressive saccades.

### Data analysis

The data obtained were submitted to statistical tests that assumed a normal sample distribution. Parametric tests were used because the violation of the normality assumption for samples over 30 is considered unproblematic (Elliott & Woodward, 2007; Ghasemi & Zahediasl, 2012; Pallant, 2001). Cronbach’s alpha was used to analyze the internal consistency of the tasks. In addition, Fleiss’ kappa (Landis & Koch, 1977; Zapf, Castell, Morawietz, & Karch, 2016) was used to assess the inter-rater reliability of the semantic decision task and to confirm the validity of the task. The kappa was calculated with six coders. The coders have experience in the area of neuropsychological assessment and were instructed on the definitions of ambiguous sentences, sentence with ARS, or sentences with ARO before evaluating the semantic decision task. Repeated measures ANOVAs were used to compare the three categories of words and sentences (regular words, pseudo-words, and quasi-words; ambiguous, subject action-related, or object action-related sentences) and their positions in sentences (subject, object, or second sentence). Effect sizes were reported in partial eta-squared, and we calculated their magnitude according to the multiple regression magnitudes (i.e., small < .03, medium < .14, large < .27; Cohen, 1988; Cohen, Cohen, West, & Aiken, 2003; Field, 2009; Watson, 2017). Additionally, stepwise linear regressions were used to identify the factors relevant to semantic decision task efficiency, correct percentage, and average time.

## Results

### Adult Dyslexia Checklist

The ADC showed a normal distribution (skewness = 0.45, kurtosis = − 0.193, Shapiro-Wilk’s test of normality = .416, n.s.). Participants’ scores on the checklist ranged from 0 to 11 points. The mean score was 4.27, and the standard deviation was 2.68. Five participants scored two points, and five participants scored five points.

### Lexical decision task

Analyses of the reliability revealed an adequate value of Cronbach’s alpha (*α* = .75). The participants answered 95.67% (S.D. = 2.63) of the lexical decision task items correctly. Pseudo-words elicited the highest percentage of correct responses (*M* = 99.49%, S.D. = 1.47), followed by regular words (*M* = 96.34%, S.D. = 2.82), and quasi-words (*M* = 93.94%, S.D. = 4.10). Specifically, the participants had an average of 7.5 errors for the 108 quasi-words and 2.4 errors for the 72 regular words. Thus, the three word types showed significant differences, *F*(2) = 36.076, *p* < .001, and the effect had a large magnitude, *η*_p_^2^ = .530.

The average trial time for each word was 1279.76 ms (S.D. = 473.31). The participants had the quickest judgment times for regular words (*M* = 1104.65, S.D. = 267.64), followed by pseudo-words (*M* = 1241.22, S.D. = 601.03), then quasi-words (*M* = 1409.36, S.D. = 597.87). Participants made decisions about regular words 300 ms faster than quasi-words and 100 ms faster than pseudo-words, and average trial times differed significantly between the three word types, *F*(2) = 12.180, *p* = .001, with a large magnitude effect, *η*_p_^2^ = .282.

Participants’ IES, which reflected their efficiency in this task, was 1342.60 ms (S.D. = 507.74). They were most efficient in identifying regular words (*M* = 1149.28 ms, S.D. = 277.25), followed by pseudo-words (*M* = 1249.80 ms, S.D. = 609.22), and then quasi-words (*M* = 1513.02 ms, S.D. = 675.48). Hence, the regular words were processed more efficiently at 363.74 ms then quasi-words. This indicates that the participants made judgments faster or more accurately when judging regular words. Efficiency also varied for each word, *F*(2) = 14.946, *p* < .001, and the effect had a large magnitude, *η*_p_^2^ = .325.

### Semantic decision task

Reliability analyses showed an excellent value of Cronbach’s alpha (*α* = .93), and Fleiss’ kappa revealed substantial agreement between the coders (Fleiss’ *Κ* = .76; observed agreement = .85; expected agreement = .36). A comparison of the three types of sentences in the semantic decision task is shown in Table 1. The participants correctly answered 81.21% (S.D. = 14.85) of the ambiguous phrases. They made the fewest misjudgments with sentences with actions related to subjects. There were no significant differences in the correct percentage between the types of phrases.

**Table 1:** Comparison of phrase types and their means

	Ambiguous	Action related to subject	Action related to object				
Measures	Mean (S.D.)	Mean (S.D.)	Mean (S.D.)	*F*(2)	*p*	*η*_p_^2^	Post hoc LSD
Correct percentage	79.3 (21.4)	85.0 (16.11)	80.91 (15.69)	1.329	.268	.040	–
Average time	4173.14 (1394.36)	4119.69 (1039.79)	4191.60 (1407.68)	0.163	.850	.005	–
IES	6216.44 (4597.33)	5142.44 (2094.53)	5459.93 (2367.42)	1.359	.255	.041	–
Average number of fixations	11.96 (5.08)	11.65 (4.27)	11.75 (4.79)	0.392	.644	.012	–
Average time per fixation	223.16 (69.21)	222.49 (65.82)	226.97 (70.39)	1.239	.297	.037	–
Percentage of regressive saccades	13.84 (3.35)	14.84 (4.06)	14.50 (3.94)	3.449	.038	.097	AMB × ARS

On average, the participants spent 4164.39 ms (S.D. = 1247.99) assessing the sentences in this task. The average time they spent did not differ significantly by sentence type.

We calculated the IES using the time spent and the percentage of correct responses. The participants’ IES was 5364.65 ms (S.D. = 2011.53) on the task. There were no significant differences between the sentence types.

In each sentence, the participants had an average of 11.83 (S.D. = 4.67) fixations. No significant difference was observed among the sentence types. Additionally, these fixations had an average duration of 223.95 ms (S.D. = 67.95).

Finally, the participants regressed 13.44% (S.D. = 4.76) of the times they performed a saccade. This means that for every 9 saccades, the participant had approximately 1 regressive saccade. We analyzed the percentage of regressive saccades by sentence type and found significantly higher percentages in sentences with subject-related actions than in ambiguous sentences or those with object-related actions. Post hoc tests revealed a significant difference between phrases that included an action related to the subject and ambiguous sentences. This effect had a medium magnitude, *η*_p_^2^ = .097.

We used another repeated measures ANOVA to determine where the percentage of regressive saccades was highest in the different types of sentences. No significant differences were found between the subject and the object locations. However, the percentage of regressive saccades was significantly higher in the second sentence of phrases with subject-related actions than for ambiguous sentences or object-related action phrases. Post hoc tests revealed a significant difference between phrases where the action was related to the subject and ambiguous sentences. The effect size had a medium magnitude, *η*_p_^2^ = .119. The descriptive statistics can be found in Table 2.

**Table 2:** Comparison of regression locations in phrase types and their means

	Ambiguous	Action related to subject	Action related to object				
Location	Mean (S.D.)	Mean (S.D.)	Mean (S.D.)	*F*(2)	*p*	*η*_p_^2^	Post hoc LSD
Subject (%)	0.15 (0.20)	0.12 (0.31)	0.12 (0.31)	.194	.784	.006	–
Object (%)	2.80 (1.80)	2.73 (1.87)	2.71 (2.06)	.092	.912	.003	–
First sentence (%)	2.95 (1.83)	2.85 (1.98)	2.83 (2.06)	.152	.859	.005	–
Second sentence (%)	10.13 (3.52)	11.09 (3.76)	10.81 (4.45)	4.327	.017	.119	AMB × ARS

### Predicting semantic decision task efficiency based on lexical measures

To predict semantic decision task efficiency, a stepwise linear regression with all the lexical measures (average time and correct percentage for each type of word) was used as the independent variable, and the semantic IES was used as the dependent variable. This generated one model (multiple *R* = .51) with the average time for regular words as the only predictor. This predictor could explain 24% of the variation in the IES. Table 3 shows the coefficients of the two regressions.

**Table 3:** Linear regression for semantic IES and its coefficients

	Beta	*t*	Sig.	Correlation coefficients			Tolerance
				Zero-order	Partial	Semi-partial	
Semantic IES model (*R*^2^ = .26; adjusted *R*^2^ = .24)							
Avg. time on reg. words	.512	3.261	.003	.512	.512	.512	1.000

To understand these results, we conducted additional regressions. The first used the same independent variables and correct percentages for the semantic decision task as the dependent variable. No models were created. The second again used the same independent variables but used the average time on the semantic decision task. Three models emerged: the first (multiple *R* = .73) had the average time on quasi-words as the only predictor; the second (multiple *R* = .76) had the average time on quasi-words and average time on pseudo-words as the predictors; and the last (multiple *R* = .82) had the average time on quasi-words, the average time on pseudo-words, and the correct percentage of regular words as predictors. The predictor of the first model could predict 52% of the variation in the average time, the variables of the second model could explain 57%, and the variables in the third model could explain 63% of the variance. The coefficients of the regressions are presented in Table 4.

**Table 4:** Linear regression for semantic average time and its coefficients

	Beta	*T*	Sig.	Correlation coefficients			Tolerance
				Zero-order	Partial	Semi-partial	
Semantic avg. time model 1 (*R*^2^ = .54; adjusted *R*^2^ = .52)							
Avg. time on quasi-words	.732	5.893	.000	.732	.732	.732	1.000
Semantic avg. time model 2 (*R*^2^ = .60; adjusted *R*^2^ = .57)							
Avg. time on quasi-words	1.639	3.759	.001	.732	.572	.441	.072
Avg. time on pseudo-words	− 0.941	− 2.158	.039	.637	− .372	− .253	.072
Semantic avg. time model 3 (*R*^2^ = .67; adjusted *R*^2^ = .63)							
Avg. time on quasi-words	1.710	4.195	.000	.732	.621	.459	.072
Avg. time on pseudo-words	− 0.958	− 2.356		.637	− .407	− .258	.072
Correct pct. on reg. words	− 0.259	− 2.311	.026 .028	− .087	− .400	− .253	.955

The ADC score was also used to predict semantic efficiency, but no models were formed.

## Discussion

The aim of this study was to identify the relevant lexical factors in a lexical decision task for a semantic decision test. To accomplish this goal, we used linear regressions with the lexical decision task measures to predict the semantic task efficiency.

The lexical decision task revealed an adequate index of reliability. In addition, this task has been extensively used and validated in previous research (see Araújo et al., 2015; Haro et al., 2017; Hicks et al., 2017; or Oliveira, 2014; Oliveira & Justi, 2017 for studies using lexical decision tasks in Portuguese). In the lexical decision task, the correct percentages of regular words and quasi-words were higher than those found by Oliveira (2014), who reported correct percentages of 89.16% (S.D. = 5.78) for regular words and 84.93% (S.D. = 8.53) for quasi-words. No such differences were found for pseudo-words. Oliveira (2014) found a correct percentage of 97.44% (S.D. = 2.34), which was expected because these words do not exist, so university students should not have had any major problems. In contrast, the average time in the trial was much faster than that in Oliveira’s study. The slowest category was quasi-words, which showed a reaction time of 808.57 ms (S.D. = 156.55). The fastest category was pseudo-words. It is possible to understand that the difference in correct percentages between these two variables as a speed-accuracy tradeoff: People who read quickly will lose accuracy and vice versa (Heitz, 2014). Additionally, the difference can be explained by the fact that we used only 216 words in our study, and Oliveira used more than twice that amount. Other studies used similar paradigms in the lexical decision task (Araújo et al., 2015; Oliveira & Justi, 2017), and studies with adults found a similar percentage (95%) and a similar reaction time (917 ms, S.D. = 164).

The semantic decision task revealed adequate indexes of reliability and validity. Adopting the benchmarks of Gwet (2012), we confirmed the inter-rater agreement was high and the strength of agreement was excellent. Interestingly, the semantic decision task did not show significant differences between the three types of sentences, with the exception of the regressive saccades. We expected no differences in reaction times because although there is an extra cost involved in processing ambiguous lexical items, there is no evidence that processing differs between the reading of syntactically ambiguous sentences and the reading of unambiguous sentences (Clifton et al., 2007). The garden path model (Frazier, 1987; Frazier & Rayner, 1982; Gazzaniga et al. 2002) explains that when we have ambiguous sentences, we tend to go through the path with the fewest nodes: In our task, this leads to actions related to objects. If an action is related to the subject and the reader notices that the second sentence does not match an action related to the object, he or she will regress more frequently than in the other types of sentences to confirm the mismatch. Additionally, inter-word regressions are expected when the reader experiences comprehension difficulty (Vitu, 2013). These results are particularly interesting because they were the only significant measurement distinctions in this task. None of the other measures were significantly different but the percentage of regressive saccades: This indicates the importance of using eye-tracking recording devices in reading studies.

We also found that the phrases with subject-related actions triggered more regression in the second sentence. This finding may be misleading given the expectation that participants regress to the subject of the first sentence. Participants do not necessarily need to return to the subject to confirm whether the second sentence relates to it. Instead, they will apply a regressive pattern to locate any available information to confirm their assumption (Frazier & Rayner, 1982; Vitu, 2013). In our task, the closest such information available was in the second sentence, which explains why regressions more frequently occurred there.

In relation to the participants, they scored low on the ADC, which means they showed few signs of dyslexia. Additionally, although the participants were all right-handed, there is no evidence that handedness is a confounding factor for reaction time measures. It is important to note that they were faster judging regular words (which they pressed the button with their left hand). For this reason, it is assumed that dominant hand use had no confounding effect.

A linear regression revealed that the average time on regular words in the lexical decision task predicted efficiency in the semantic decision task. Word recognition speed is correlated to vocabulary size (Fernald et al., 2006). Additionally, vocabulary and lexical depth can predict reading comprehension (Perfetti & Stafura, 2014), which explains why the average time spent on regular words predicted efficiency on the semantic decision task.

From these results, we can confirm that the lexical decision task can predict only a portion of semantic decision task efficiency. The lexical component can predict approximately 24% of semantic decision efficiency. Our findings are consistent with those of other studies. Swart et al. (2017) attempted to predict a mean of several measures of a semantic task from several variables related to the lexicon. He also used other measures, such as reasoning and decoding, and was able to explain 65% of the variation in the semantic tasks, but only 30% of the variation was related to lexical measures. Although we only used lexical decision task measures, we were able to predict roughly the same amount, and we were able to infer that the portion that we were not able to predict may be related to non-verbal reasoning and decoding. Other studies were able to predict semantic comprehension with vocabulary measures, but not much more than our study (Ouellette, 2006), and others were able to predict 6.1 to 11.9% of the comprehension measures with tasks similar to the lexical decision task (Cutting & Scarborough, 2006). All of these studies were performed with elementary school students. Thus, our findings are consistent with those of other studies and suggest that the lexicon is able to predict only 10–30% of semantic comprehension.

We expected greater predictive powers, but our results may be explained by the nature of the lexical decision task. Rayner and Liversedge (2013) stated that this type of task can be limited in relation to the processing of word identification. The study of isolated words may oversimplify the reading process because reading an isolated list of words is atypical in normal reading. Additionally, it may fail to deliver visual information about the words to the lexical processing system. In normal reading, visual and orthographic information is first accessed in the parafovea and processed at superficial levels before the attention is shifted to it. This causes the words to be processed at multiple levels. The pattern of fixations in reading will determine the quality and the quantity of orthographic information that will be processed, but all of this is lost when only a list of words is shown. Other studies point in the same direction, demonstrating that lexical decision tasks are not a good measure of lexical access (Balota & Chumbley, 1984).

In addition, this type of task contains a high degree of noise (Diependaele et al., 2012). Finally, Cutting and Scarborough (2006) suggest that these types of tasks may evaluate different cognitive processes. Therefore, this paradigm may be only partially connected with the semantic decision task.

Although this task may not be ideal for predicting the reading process, it is a useful paradigm for other types of research. The use of this task has been increasing and has been used to understand priming (Oliveira & Justi, 2017), event-related potentials (Araújo et al., 2015; Haro et al., 2017), memory (Hicks et al., 2017), and several other phenomena.

From our results, we can conclude that reading efficiency relates to lexical processes. Previous research has suggested this connection (Hall, Greenberg, Laures-Gore, & Pae, 2014; Swart et al., 2017), but our study is one of the first to examine a direct connection between lexical processes and efficiency. Also, this is one of the first to find a relationship between reading comprehension and lexical processes in adults. The implications of our findings are also practical. For instance, we can theorize that interventions in the lexicon and in expanding the depth of the lexical knowledge will result in better reading efficiency. With such interventions, those with vocabulary problems and reading difficulties should be able to perform better in both areas, even if intervention takes place in only one of them.

It is important to note that our principal measure of the semantic task was the IES (Bruyer & Brysbaert, 2011). We chose this measure because we wanted to assess reader efficiency, not just their reading speed or accuracy; thus, we wanted to look for efficient (fast and accurate) readers. The choice of this variable was thus consistent with the purpose of this study. If we had used only the percentage of correct responses, we would not have been able to generate a model in the regression. Other studies (see Ouellette, 2006; Swart et al., 2017) used the mean score of various semantic tasks to create a “semantic variable.” The prediction of the IES in our study was similar to the mean scores in other studies. Thus, we strongly encourage the use of the IES for other studies with similar tasks and objectives.

In future studies, it would be interesting to evaluate the semantic score from a multi-faceted perspective, since the comprehension process is complex, and some tests may measure different abilities (Keenan et al., 2008). It is thus necessary to determine if the relation found in this study can be found in other measures of reading comprehension. One such measure could be tests of complex reading, such as banked gap-fill tasks (Mccray & Brunfaut, 2018). Other future studies should also focus in interventions. We theorize that interventions that seek to improve the lexicon should also result in better reading efficiency. It is important to determine if this connection also appears in elementary school students and in adults with reading or vocabulary difficulties.

Some of the limitations of the present study were that our sample was composed mainly of university students who read frequently, making them atypical of the surrounding population, since the Brazilian reading standard has a high index of functional illiteracy (INAF, 2016). Other limitations were that our semantic decision task was not validated, and we only used one semantic measure. Thus, we encourage other researchers to use larger and more diverse samples. It would also be interesting to attempt to validate the semantic decision task.

## Conclusions

To conclude, the aim of the study was to understand which lexical factors in a lexical decision task are relevant in a semantic decision test. We found that the average time spent on words predicted 24% of efficiency. We expected a larger percentage, but this result may be explained by the nature of the lexical decision task, which questions this paradigm of lexical access. Finally, inter-word regressions were the only significant measure in our semantic decision test when comparing the three types of sentences.

## Abbreviations

ADC: Adult Dyslexia Checklist
AMB: Ambiguous phrases
ARO: Unambiguous sentences with actions related to the object
ARS: Unambiguous sentences with actions related to the subject
IES: Inverse efficiency score
JPEG: Joint Photographics Experts Group
SMI: SensoMotoric Instruments
